# Detection and Prediction of Toxic Aluminum Concentrations in High‐Priority Salmon Rivers in Nova Scotia

**DOI:** 10.1002/etc.5997

**Published:** 2024-10-01

**Authors:** Kristin A. Hart, Benjamin Trueman, Edmund A. Halfyard, Shannon M. Sterling

**Affiliations:** ^1^ Hydrology and Climate Change Research Group, Department of Earth and Environmental Sciences Dalhousie University Halifax Nova Scotia Canada; ^2^ Department of Civil and Resource Engineering, Centre for Water Resources Studies Dalhousie University Halifax Nova Scotia Canada; ^3^ Nova Scotia Salmon Association Bedford Nova Scotia Canada

**Keywords:** Atlantic salmon, Environmental chemistry, Environmental modeling, Inorganic aluminum, Metal speciation, Metal toxicity, Water quality

## Abstract

Elevated concentrations of toxic cationic aluminum (Al_i_) are symptomatic of terrestrial and freshwater acidification and are particularly toxic to salmonid fish species such as Atlantic salmon (*Salmo salar*). Speciated metal samples are rarely included in standard water monitoring protocols, and therefore the processes affecting Al_i_ dynamics in freshwater remain poorly understood. Previous analysis of Al_i_ concentrations in Nova Scotia (Canada) rivers found that the majority of study rivers had concentrations exceeding the threshold for aquatic health, but a wide‐scale survey of Al_i_ in Nova Scotia has not taken place since 2006 (Dennis, I. F., & Clair, T. A., 2012, *Canadian Journal of Fisheries and Aquatic Sciences, 69*(7), 1174–1183). The observed levels of dissolved aluminum in Atlantic salmon (*Salmo salar*) rivers of Atlantic Canada have potential serious and harmful effects for aquatic populations. We present the findings of the first large‐scale assessment of the Al_i_ status of Nova Scotia rivers in 17 years; we measured Al_i_ concentrations and other water chemistry parameters at 150 sites throughout the Southern Uplands region of Nova Scotia from 2015 to 2022. We found that Al_i_ concentrations exceeded toxic thresholds at least once during the study period at 80% of the study sites and that Al_i_ concentrations increased during the study period at all four large‐sample study sites. Modeling of relationships between Al_i_ concentrations and other water chemistry parameters showed that the most important predictors of Al_i_ are concentrations of the dissolved fractions of Al, iron, titanium, and calcium, as well as dissolved organic carbon and fluoride. We developed a fully Bayesian linear mixed model to predict Al_i_ concentrations from a test data set within 15 μg/L. This model may be a valuable tool to predict Al_i_ concentrations in rivers and to prioritize areas where Al_i_ should be monitored. *Environ Toxicol Chem* 2024;43:2545–2556. © 2024 The Author(s). *Environmental Toxicology and Chemistry* published by Wiley Periodicals LLC on behalf of SETAC.

## INTRODUCTION

Cationic aluminum (Al_i_) is toxic to salmonids, and increased concentrations of these aluminum (Al) species are one of the most lethal effects of terrestrial and freshwater acidification. Geology is a major determinant of stream water quality within a drainage basin. The third most common element on the surface of the earth is Al, making it a common constituent of surface water chemistry. It can be toxic to aquatic organisms in circumneutral waters (Gensemer & Playle, 1999), with the Al_i_ species, such as Al^3+^, Al(OH)_2_^1+^, and Al(OH)^2+^, considered the most labile and therefore the most toxic. These positively charged Al species bind to negatively charged fish gills and cause morbidity and mortality through suffocation (Exley et al., 1991), reduce nutrient intake at gill sites, and alter blood plasma levels (Nilsen et al., 2010). The effects of sublethal exposure to freshwater Al elicit osmoregulatory impairment (Monette & McCormick, 2008; Regish et al., 2018), which reduces survival in the hypertonic marine environment (McCormick et al., 2009; Staurnes et al., 1996).

Burning of fossil fuels has resulted in the acidification of soils and surface waters during the last century through acid deposition (see Kerekes et al., 1986), which has led to increased concentrations of toxic Al_i_ in soils and drainage waters. Following reductions in anthropogenic sulfur emissions in North America and Europe, the acidification problem was widely considered solved. Many studies observed steady improvements in stream chemistry (Evans et al., 2001; Monteith et al., 2014; Skjelkvåle et al., 2005; Stoddard et al., 1999; Warby et al., 2005), including reduced concentrations of Al in the United States (Baldigo & Lawrence, 2000; Buchanan et al., 2017; Burns et al., 2006) and Europe (Beneš et al., 2017; Davies et al., 2005; Monteith et al., 2014). However, recent evidence highlights delayed recovery from acidification in certain regions with slow‐weathering geology (Houle et al., 2006; Warby et al., 2009; Watmough et al., 2016), including Nova Scotia, Canada (Clair et al., 2011; Sterling et al., 2020). This raises questions about the possibility of elevated and/or increasing Al concentrations in freshwater systems.

Previous research has shown widespread increases in total Al (Al_t_) in Nova Scotia (S. Sterling et al., unpublished data); however, a knowledge gap exists as to which species of Al are driving these trends—Al_i_ or the less toxic organically complexed Al (Al_o_). The concentration at which Al_i_ becomes toxic to aquatic organisms is dependent on pH, temperature, ionic strength, concentrations of base cations, and the presence of dissolved organic matter (Gensemer & Playle, 1999). Some of this complexity has been recently addressed by advanced modeling, such as biotic ligand models (Santore et al., 2018), but it remains difficult to select a single threshold to guide interpretation of data and the creation of regulatory limits. In the present study we assume a toxic threshold of 15 μg/L, which Kroglund et al. (2007) showed negatively impacts anadromous Atlantic salmon in acidic conditions similar to those of our Nova Scotia study. An earlier study of Al concentrations in Nova Scotia led to the discovery that concentrations of Al_i_ in Nova Scotia rivers currently exceed this assumed toxic threshold for aquatic health of 15 μg/L (Dennis & Clair, 2012); however, no studies of Al_i_ concentrations in Nova Scotia have been sufficiently long term to determine speciated temporal trends.

The processes that affect Al_i_ dynamics remain poorly understood due to limited sampling of speciated Al. Speciated sampling can be time‐consuming and expensive, and is not often included in regular water chemistry monitoring programs (Driscoll & Schecher, 1990). Speciation of Al is complex and mostly determined by pH and the presence or absence of organic material (typically measured as dissolved organic carbon [DOC]; Santore et al., 2018). The pH is generally negatively correlated with Al_i_ concentrations (Campbell et al., 1992; Kopáček et al., 2006; Kroglund et al., 2001; Seip et al., 1989; Teien et al., 2006), because lowered pH increases the solubility of secondary minerals containing Al. Previous studies have also shown that Al concentrations are positively correlated with DOC concentrations (Campbell et al., 1992; Kopáček et al., 2006). Previous modeling has shown that DOC and water temperature (*T*_w_) are the most significant predictors of Al_i_ concentrations (Sterling et al., 2020), but this model needs to be improved with a larger sample size. The previous model was built from a data set containing 10 sites with 5 to 47 samples collected at each site.

Several programs have been developed to model aqueous Al_i_ concentrations, such as ALCHEMI, WHAM, and Visual MINTEQ (see Cory et al., 2007; Sjöstedt et al., 2010; Tangen et al., 2002). These models are based on the thermodynamic relationships that control the behavior and speciation of aqueous Al (Gustafsson, 2020; Schecher & Driscoll, 1995; Tipping, 1998). While the thermodynamic constants for inorganic complexes are typically well understood, reactions involving organic complexation are more difficult to quantify and can lead to poor precision and considerable uncertainty in chemical equilibrium modeling (Driscoll & Schecher, 1990; Schecher & Driscoll, 1995; Sjöstedt et al., 2010).

We aimed to complete the first large‐scale assessment of Al_i_ concentrations with repeated measurements in Nova Scotia. We built on a one‐sample large‐scale survey in 2006 (Dennis & Clair, 2012), to detect temporal trends in speciated Al concentrations as well as to determine potential predictors of Al_i_ concentrations and use these to build a simple empirical model to increase our understanding of the factors affecting Al_i_ dynamics in dilute, acidified freshwaters.

## MATERIALS AND METHODS

### Study area

**Figure 1:**
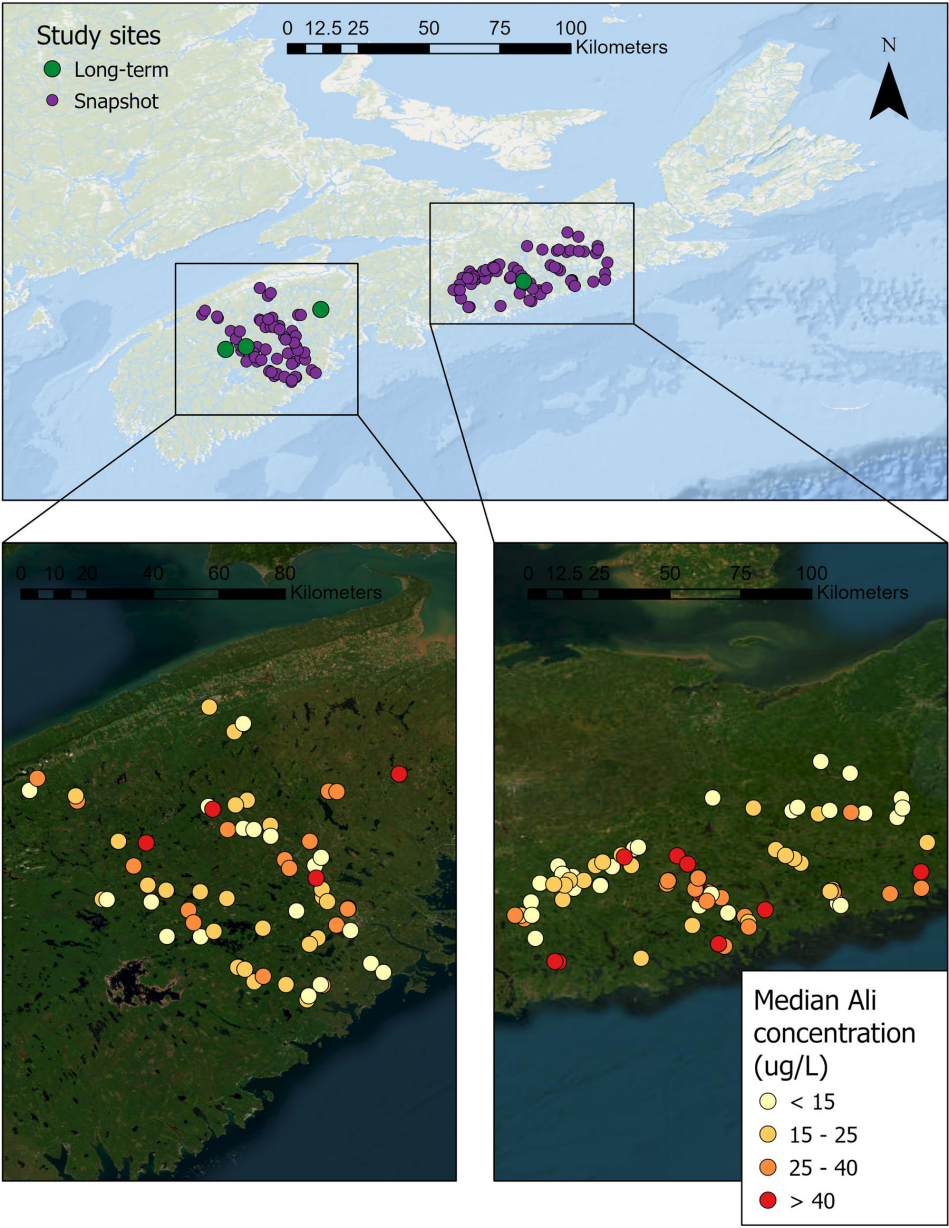
Locations of large‐ and small‐sample sites and median Al_i_ concentration at each site during the study period (2015–2022).

We surveyed Al_i_ and other water chemistry parameters at 150 sites in 18 watersheds across the Southern Uplands region of Nova Scotia (Figure 1). Large‐sample (2015–2022) water chemistry measurements were conducted at four catchments: Mersey River, Moose Pit Brook, Maria Brook, and Brandon Lake Brook (Table 1). A “snapshot” of the water chemistry conditions was captured at the remaining sites, with 1 to 54 samples being collected at each site between 2020 and 2022. The four major large‐sample study catchments and the majority of the “snapshot” sites are predominantly forested and drain slow‐weathering, base‐cation poor bedrock, producing soils with a low acid‐neutralizing capacity (Langan & Wilson, 1992; Tipping, 1989). The catchments also have relatively high aquatic DOC concentrations (Ginn et al., 2007) associated with the abundant wetlands in the region (Clair et al., 2008; Gorham et al., 1986; Kerekes et al., 1986). Due to this combination of characteristics, the study rivers are acidic (mean pH of 4.9), dilute (mean specific conductance (SPC) of 38 μS/cm), and have relatively high levels of organic acidity (mean DOC concentration of 11 mg/L).

**Table 1:** Study site characteristics for large‐sample study sites

Site	Watershed area (km^2^)	Temporal range of samples	No.	Dominant bedrock type
MB	0.47	2016–2021	32	Granite
BLB	1.4	2016–2022	41	Sandstone/slate
MPB	15.8	2015–2021	57	Granite/slate
MR	292.8	2015–2021	61	Granite

BLB = Brandon Lake Brook; MB = Maria Brook; MPB = Moose Pit Brook; MR = Mersey River.

### Data collection and analysis

We measured Al_i_ concentrations and other water chemistry parameters at the sample locations, including pH, *T*_w_, SPC, and concentrations of constituents such as dissolved metals and DOC. Measurements were taken throughout the year from 2015 to 2021 at Mersey River, Moose Pit Brook, Maria Brook, and Brandon Lake Brook, and measurements were taken from March to December during 2020 to 2022 at all other sample locations. Most study rivers were at least partially ice covered during the winter months.

Sampling events comprised grab samples for laboratory analysis and in situ measurements of pH, *T*_w_, and SPC. We calculated Al_i_ as the difference between dissolved Al (Al_d_) and Al_o_, following Dennis and Clair (2012), Tangen et al. (2002), and Sterling et al. (2020; Equation 1). Speciating metals samples in the field reduces errors caused by changes in temperature and pH during transport from field to laboratory.

(1) $${\text{Al}}_{i}={\text{Al}}_{d}-{\text{Al}}_{o}$$

We measured Al_d_ as the Al concentration of a sample passed through a 0.45‐μm polyethersulfone filter. We measured Al_o_ as the eluate from passing water through a 3‐cm negatively charged cation exchange column (Bond Elut Jr. Strong Cation Exchange Column). The columns were preconditioned with 30 mL of 0.4 mol/L ammonium acetate buffer at pH 5, followed by a rinse with 30 mL of sample water, following previous studies (Dennis & Clair, 2012; Tangen et al., 2002). Water was passed through the cation exchange column at a rate of less than 60 drops/min, to avoid underestimating Al_i_ (Tangen et al., 2002). From this method, Al_o_ was operationally defined as the nonlabile, organically complexed species of Al, and Al_i_ was defined as the cationic species of Al (e.g., Al^3+^, Al(OH)_2_^1+^, Al(OH)^2+^). This speciation process was carried out in the field.

Samples to be analyzed for metal content as just described were collected using sterilized polyethylene syringes into sterilized polyethylene tubes (15 mL). All metal samples were filtered in the field and preserved with nitric acid (HNO_3_) within 24 h of arriving at the laboratory. Samples analyzed for DOC, anion content, and physical parameters were not filtered in the field and were collected in sterilized amber glass or polyethylene bottles (1 L). All samples were kept cooled to a temperature of 4 °C during transport to the laboratory and were delivered within 48 h of being collected. Laboratory analytical methods are outlined in the Supporting Information, Appendix A.

Some samples analyzed for organic carbon content were not filtered in the laboratory; however, the suspended loads in rivers with similar characteristics to the study rivers are typically very low, and it has been established that particulate matter typically contributes <5% of total organic carbon (TOC; Clair et al., 2008; Laudon et al., 2001). We therefore used TOC as a proxy for DOC in these cases, as has been done in previous freshwater Al modeling studies (see Cory et al., 2007). All samples analyzed for organic carbon content are grouped here and reported as DOC.

In situ measurements of pH, *T*_w_, and SPC were taken using a portable water chemistry sonde (YSI ProQuatro). The sonde was calibrated at a minimum of once a week, typically the day before samples were collected.

Temporal trends were detected using the Mann–Kendall trend test from the R package “Kendall” (McLeod, 2022), which tests for monotonic trends in time series based on the Kendall rank correlation. Time series plots were smoothed using the locally estimated scatterplot smoother method. Independent linear correlations were detected using the Kendall's tau test. These statistical analyses were carried out in R Ver. 4.1.2.

### Bayesian linear mixed modeling

We built a fully Bayesian linear mixed model (BLMM) to predict Al_*i*_ concentration. Missing and left‐censored values (nondetects) were imputed in one step during model fitting by treating them as parameters; nondetects were constrained with an upper bound equal to the detection limit. That is, each of the 4000 posterior samples included a different imputed value for each missing and left‐censored value.

We split the data into a training set, used to fit the model, and a test set, used to simulate the model's predictive performance on future observations. To avoid issues of nonindependence between the training and test sets that might negatively bias the test set error (Kapoor & Narayanan, 2023), we chose a temporal split of the training and test sets. That is, the test set comprised the most recent 20% of observations from sites with at least five observations. The remaining 80% comprised the training set.

We selected (1) variables having a Pearson correlation with Al_i_ in the training set greater than 0.1, and (2) supplementary variables used to predict the missing values in the predictors of Al_*i*_ (see the equation set following). We excluded variables that were >50% missing or censored. The following variables were selected as predictors: Al_*d*_, DOC, color, pH (sonde), temperature (sonde), sulfate (SO_4_), alkalinity, fluoride (F), and dissolved fractions of calcium (Ca_d_), titanium (Ti_d_), iron (Fe_d_), cerium (Ce_d_), and lithium (Li_d_). All variables were scaled to have zero mean and unit standard deviation.

The i Al_i_ concentrations, *y*_i_, were predicted using the following equations:

Likelihood:

$$y_{i}∼T(\mu_{i},\sigma ,\nu )$$

Model for $\mu_{i}$:

$$\mu_{i}=\alpha_{{\text{site}}_{j}}+X\beta$$

Model for missing values:

$$p\sim T(\mu_{p},\sigma_{p},v_{p})\;\mathrm{for}\;\mathrm{each}\;\mathrm{predictor}\;p$$

Model for $\mu_{p}$:

$${\text{Al}}_{d}=\beta_{\text{DOC}}\text{DOC}$$

$${\text{Ti}}_{d}=\beta_{{\text{Ti}}_{d}}{\text{Al}}_{d}$$

$$\mathrm{DOC}=\alpha_{\text{DOC}}$$

$$\mathrm{Color}=\beta_{\text{color}}\text{DOC}$$

$${\text{Fe}}_{d}=\beta_{{\text{Fe}}_{d}}\text{DOC}$$

$${\text{Ce}}_{d}=\beta_{{\text{Ce}}_{d}}{\text{Fe}}_{d}$$

$${\text{pH}}_{\text{sonde}}=\beta_{\mathrm{pH}}{\text{Al}}_{d}$$

$${\text{Ca}}_{d}=\alpha_{{\text{Ca}}_{d}}$$

Temperature=γ+f(t)

$$f\left(t\right)=Z_{s}b_{s}$$

$${\text{SO}}_{4}=\beta_{{\text{SO}}_{4}}{\text{Ca}}_{d}$$

$$\mathrm{Alkalinity}=\beta_{\text{Alkalinity}}{\text{Ca}}_{d}$$

$${\text{Li}}_{d}=\beta_{{\text{Li}}_{d}}F$$

$$F=\alpha_{F}$$

Priors:

$$\sigma ,\sigma_{p},\sigma_{\alpha },\sigma_{b}\sim \mathrm{Half}‐T\left(0,2.5,3\right)$$

$$v,v_{p}\sim \text{Gamma}(2,0.1)$$

$$\alpha_{{\text{site}}_{j}}\sim N\left(0,\sigma_{\alpha }\right)\;\mathrm{for}\;j\;\mathrm{in}\;1\ldots 150$$

β~N(0,τ)

τ~Half‐Cauchy(0,1)

$$\beta_{\mu_{p}}\sim \;N\left(0,1\right)$$

$$b_{s}\;\sim \;N\left(0,\sigma_{b}\right)$$

where *N* represents the Gaussian distribution with mean μ and standard deviation *σ*, *T* represents the *t*‐distribution with *μ*, *σ*, and *v* representing the degrees of freedom. The parameter $\alpha_{{\text{site}}_{j}}$ is a random intercept centered at $\bar{\alpha }$, *X* is the linear model design matrix, and β is a vector of model coefficients, centered at zero with standard deviation *τ*. The hierarchical Gaussian prior on β helps to stabilize the estimates of multicollinear predictors (Hastie et al., 2009), and also shrinks coefficient estimates toward zero, which has a similar effect to a multiple comparisons correction (Gelman et al., 2012). Supporting Information, Figure S3, in Appendix B shows the correlations between variables included in the model. In the missing value model for temperature, the function *f*(*t*) represents a cyclic cubic regression spline (Wood, 2017) with penalized coefficients $b_{s}$ and basis function matrix $Z_{s}$. The variable *t* represents ordinal day.

Missing and left‐censored values (nondetects) were modeled as parameters and sampled from the joint posterior during model fitting, along with the regression coefficients and the parameters used to predict missing values in new observations (Bürkner, 2017; McElreath, 2020). Left‐censored values were constrained to fall below the respective censoring limits.

Model code was written in Stan (Stan Development Team, 2023) using a template generated in brms (Bürkner, 2017, 2018). Several other R packages were used for data cleaning and visualization (Firke et al., 2023; Fischetti, 2022; Grolemund & Wickham, 2011; Kay, 2023; Pedersen, 2022; Ram et al., 2018; Vaughan et al., 2022; Wickham & Bryan, 2023; Wickham et al., 2019; Wilke & Wiernik, 2022).

## RESULTS AND DISCUSSION

### Status of Al_i_ concentrations in Nova Scotia

The assumed toxic threshold of 15 μg/L for Al_i_ concentrations was exceeded at 120 of the 150 study sites (80%) at least once during the study period. Median Al_i_ concentrations ranged from 1.1 to 76.3 μg/L across all sites (Figure 1), with the median concentration exceeding the toxic threshold at 99 of the 150 sites (66%). For many (~70%) of these sites, sample collection timing was limited to the window of Atlantic salmon (*Salmo salar*) smoltification (typically early April to early June). Exceedances of Al_i_ during this window would have a large negative impact on salmon smolts because they undergo rapid physiological transformation and experience the osmoregulatory demands associated with anadromy (Monette & McCormick, 2008; Staurnes et al., 1996). The median Al_i_ concentration at all four large‐sample study sites also exceeded the toxic threshold (Table 2).

**Table 2:** Median and standard deviation values for select water chemistry parameters for large‐sample study sites

Site	Variable	Median	SD
MB	Al_i_ (μg/L)^a^	44.5	28.5
	Al_o_ (μg/L)	329	110
	Al_d_ (μg/L)	364	123
	DOC (mg/L)	12.1	5.61
	Ca_d_ (mg/L)	1.60	0.366
	pH	4.65	0.618
BLB	Al_i_ (μg/L)^a^	46.4	36.7
	Al_o_ (μg/L)	362	102
	Al_d_ (μg/L)	420	111
	DOC (mg/L)	15.1	4.44
	Ca_*d*_ (mg/L)	1.12	0.293
	pH	4.43	0.452
MPB	Al_i_ (μg/L)^a^	21.0	16.2
	Al_o_ (μg/L)	247	101
	Al_d_ (μg/L)	260	111
	DOC (mg/L)	17.6	7.70
	Ca_d_ (mg/L)	0.890	0.388
	pH	4.23	0.394
MR	Al_i_ (μg/L)^a^	22.0	12.5
	Al_o_ (μg/L)	170	66.3
	Al_d_ (μg/L)	204	71.0
	DOC (mg/L)	9.00	3.48
	Ca_d_ (mg/L)	0.735	0.169
	pH	4.72	0.513

^a^ Al_*i*_ is a calculated value; all other parameters are directly measured.

BLB = Brandon Lake Brook; DOC = dissolved organic carbon; MB = Maria Brook; MPB = Moose Pit Brook; MR = Mersey River.

**Figure 2:**
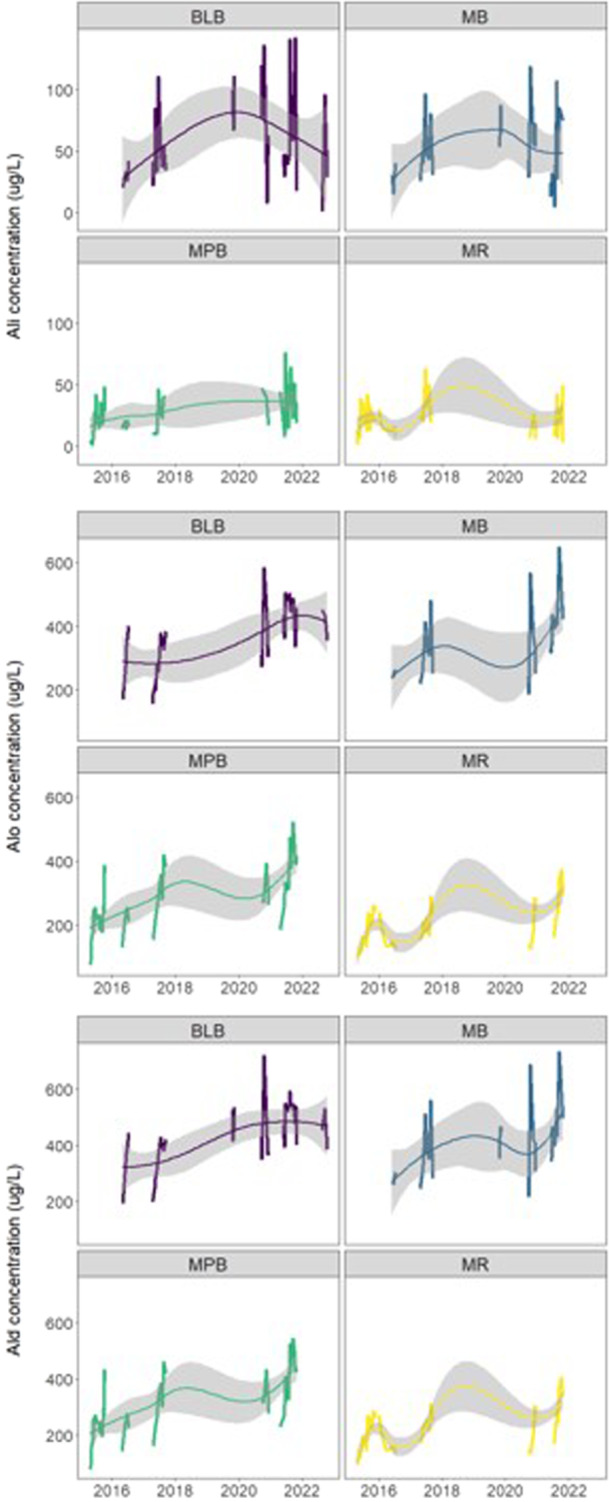
Al_i_, Al_o_, and Al_d_ concentrations at large‐sample study sites during the study period (2015–2022). Trend lines were smoothed using the locally estimated scatterplot smoother (LOESS) method. BLB = Brandon Lake Brook; MB = Maria Brook; MPB = Moose Pit Brook; MR = Mersey River.

**Figure 3:**
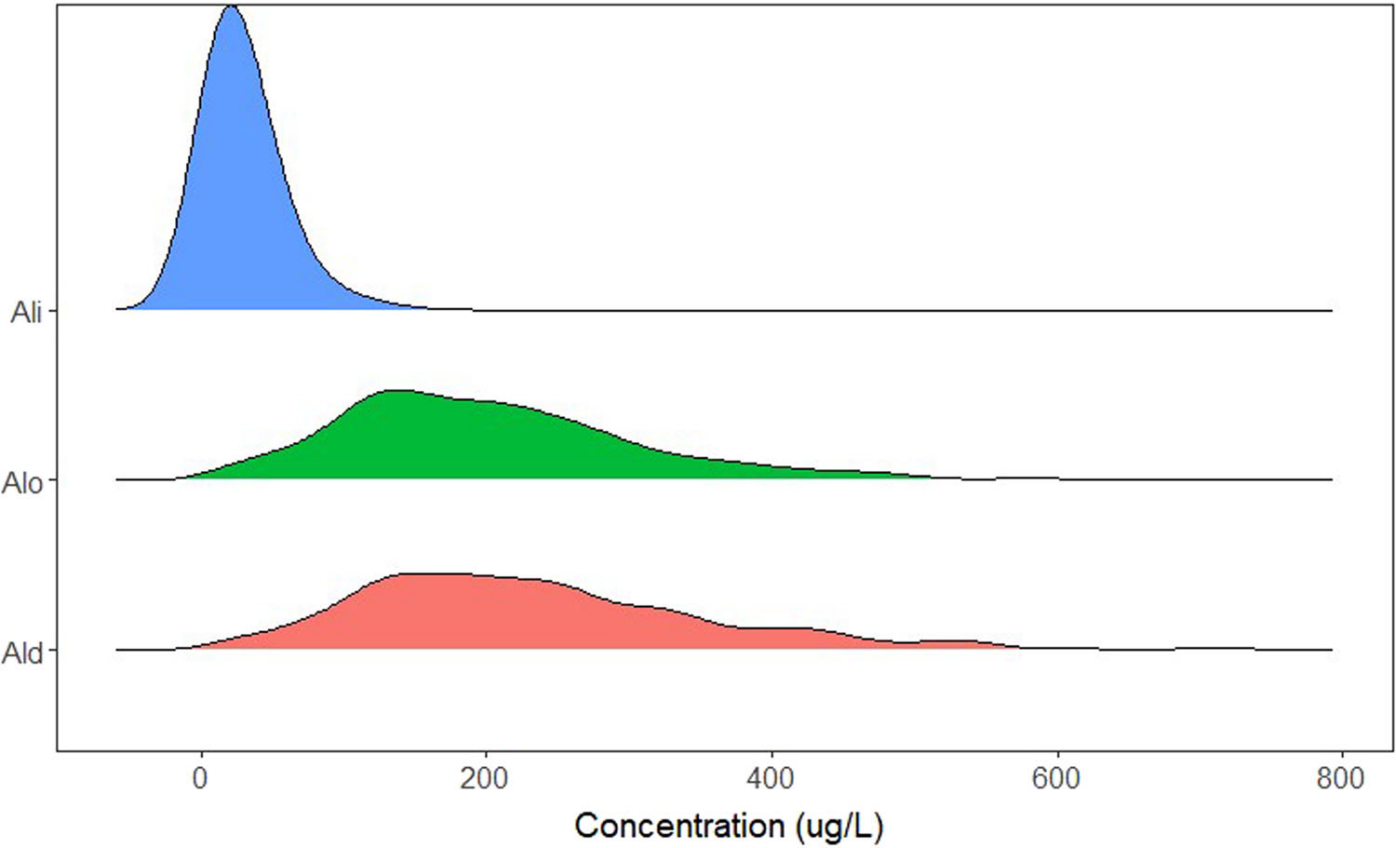
Frequency distribution plots of the concentrations of Al_i_, Al_o_, and Al_d_ at all study sites.

At the four large‐sample study sites, Al_i_ concentrations exceeded the toxic threshold in 67.2% to 97.4% of samples, depending on the site (Table 2). Over the entire large‐sample data set, Al_i_ concentrations significantly increased from the start to the end of the study period (2015–2022; Table 3). Within individual sites, Al_i_ concentration increased at all four sites; however, this increase was statistically significant (*p* < 0.05) only at one site (Moose Pit Brook; Table 3 and Figure 2). Across the entire data set and within each individual site, concentrations of Al_o_ and Al_d_ significantly increased in all cases (Table 3 and Figure 2). The only statistically significant change in the proportion of Al_*i*_ making up Al_d_ (% Al_i_) occurred at Maria Brook, where there was a decrease in this proportion (Table 3). These trends indicate that the observed trend of increasing Al_d_ concentrations is likely predominantly driven by increases in Al_o_, and visualizing the frequency distribution of the different forms of Al across all sites confirms that most of the Al_d_ at the study sites is comprised of Al_o_ (Figure 3). However, concentrations of Al_*i*_ are still increasing at all sites, and the majority of samples had Al_i_ concentrations in exceedance of the toxic threshold for salmon.

**Table 3:** Results of Mann–Kendall test for temporal trends in large‐sample study sites

Site	Variable	Tau	*p* value
All large‐sample sites	Al_i_	**0.294**	**<0.0001**
	Al_o_	**0.545**	**<0.0001**
	Al_d_	**0.533**	**<0.0001**
	%Al_i_	0.037	0.4522
BLB	Al_i_	0.184	0.0920
	Al_o_	**0.465**	**<0.0001**
	Al_d_	**0.483**	**<0.0001**
	%Al_i_	−0.058	0.5976
MB	Al_i_	0.103	0.4173
	Al_o_	**0.492**	**0.0003**
	Al_d_	**0.419**	**0.0008**
	%Al_i_	−0.004	0.9871
MPB	Al_i_	**0.357**	**<0.0001**
	Al_o_	**0.586**	**<0.0001**
	Al_d_	**0.574**	**<0.0001**
	%Al_i_	0.129	0.1582
MR	Al_i_	0.032	0.7226
	Al_o_	**0.480**	**<0.0001**
	Al_d_	**0.428**	**<0.0001**
	%Al_i_	−**0.211**	**0.0166**

Significant trends (*p* < 0.05) are bolded. Visual representations of select temporal trends are shown in Figure 2. All concentrations of aluminum species are reported in units of μg/L.

BLB = Brandon Lake Brook; MB = Maria Brook; MPB = Moose Pit Brook; MR = Mersey River.

### Potential drivers of Al_*i*_ concentrations

**Figure 4:**
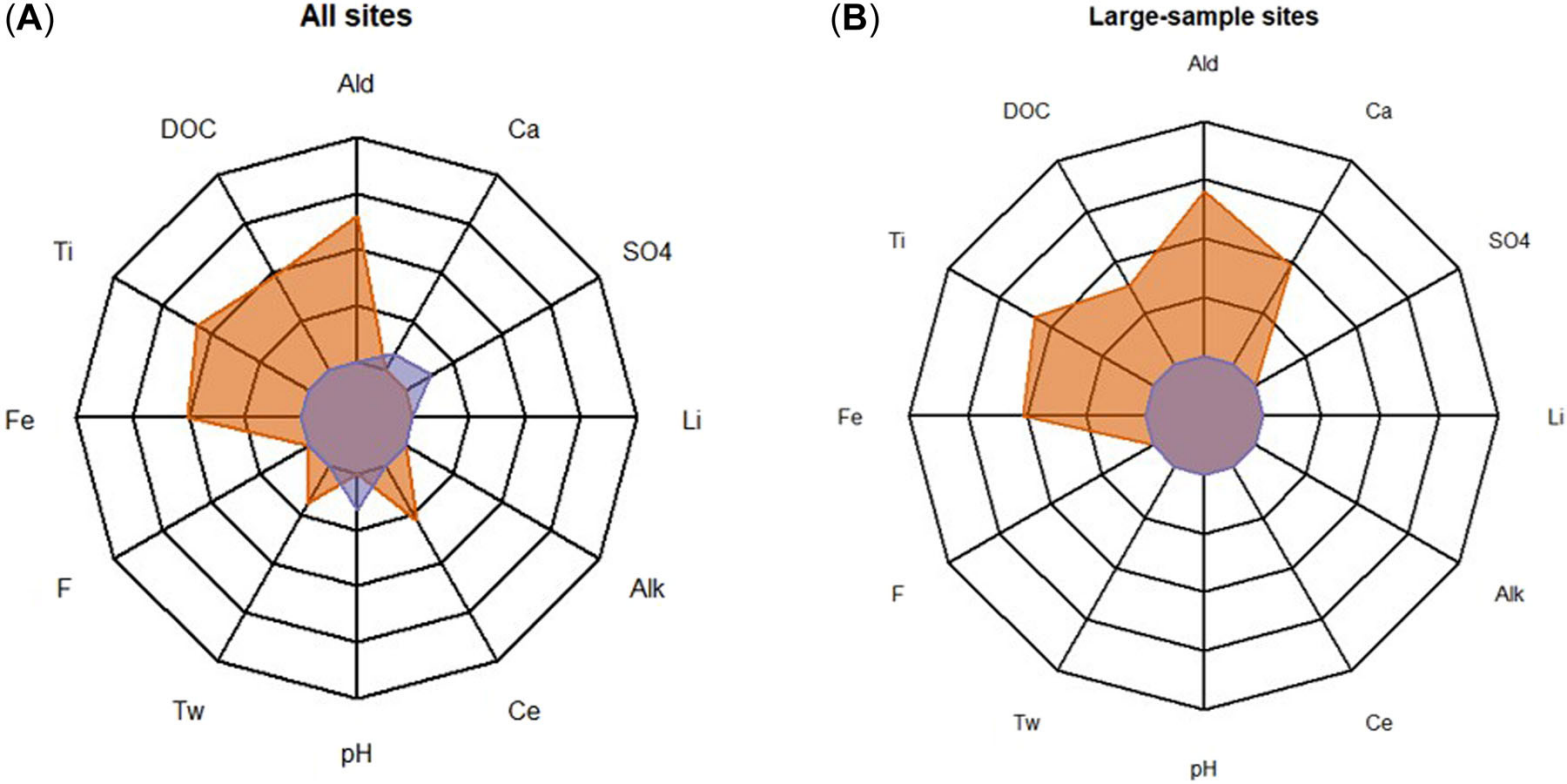
Pearson correlation values among water chemistry parameters and Al_i_ concentration at (**A**) all study sites and (**B**) only large‐sample study sites, where orange polygons indicate a positive correlation with Al_i_ and purple polygons indicate a negative correlation with Al_i_. Only correlations that are statistically significant (*p* < 0.05) are visualized. Correlation data are listed in Table 4. Alk = alkalinity; DOC = dissolved organic carbon; *T*_w_ = water temperature.

Concentrations of Al_i_ were significantly correlated with several other water chemistry parameters. When the entire database and also only the large‐sample study sites were considered, Al_i_ concentrations were strongly positively correlated with Al_d_, Ti_d_, Fe_d_, and DOC concentrations (Figure 4 and Table 4) When the entire data set was considered, there were also slight but significant positive correlations between Al_i_ concentrations and *T*_w_ and Ce_d_ concentrations; however, these correlations became nonsignificant when the data set was filtered to contain only the large‐sample study sites. Similarly, when the entire data set was considered, Al_i_ was significantly negatively correlated with SO_4_ and Ca_d_ concentrations as well as with pH; these correlations were nonsignificant when the data set was filtered to contain only the large‐sample study sites. Within only the large‐sample study sites, the correlation between Al_i_ and Ca_d_ concentrations was much stronger but unexpectedly positively correlated.

**Table 4:** Results of Pearson's test for correlation between Al_i_ concentration and various water chemistry parameters

Site	Variable	Pearson's *r*	*p* value
All sites	Al_d_	**0.651**	**<0.0001**
	Ca_d_	−**0.077**	**0.0458**
	SO_4_	−**0.130**	**0.0010**
	Li_d_	0.075	0.1048
	Alkalinity	−0.042	0.3623
	Ce_d_	**0.283**	**<0.0001**
	pH	−**0.164**	**<0.0001**
	*T*_w_	**0.188**	**<0.0001**
	F	0.041	0.3097
	Fe_d_	**0.505**	**<0.0001**
	Ti_d_	**0.571**	**<0.0001**
	DOC	**0.480**	**<0.0001**
Large‐sample sites	Al_d_	**0.702**	**<0.0001**
	Ca_d_	**0.484**	**<0.0001**
	SO_4_	0.023	0.7597
	Li_d_	−0.077	0.5276
	Alkalinity	−0.013	0.9264
	Ce_d_	0.314	0.0750
	pH	−0.022	0.7667
	*T*_w_	0.015	0.8454
	F	0.061	0.4349
	Fe_d_	**0.520**	**<0.0001**
	Ti_d_	**0.580**	**<0.0001**
	DOC	**0.384**	**<0.0001**

Significant relationships (*p* < 0.05) are bolded. A visual representation of these results is shown in Figure 4.

DOC = dissolved organic carbon; *T*_w_ = water temperature.

The positive relationship between Al_i_ and Al_d_ was expected because Al_d_ is the source of Al_i_. The positive relationship between Al_i_ and DOC has also been reported in previous studies (Campbell et al., 1992; Sterling et al., 2020), although it is widely believed that increased DOC levels protect from Al toxicity (Cardwell et al., 2018; Gensemer et al., 2018). Previous studies of metal concentrations in surface freshwaters have found good correlation between Al and Fe concentrations (Gaillardet et al., 2003; Pokrovsky & Schott, 2002), in agreement with the relationship we observed. Titanium is a tetravalent element that is generally assumed to be immobile during weathering; however, migration of Ti has been observed in organic‐ and iron‐rich rivers, and a strong positive correlation between Ti and Fe has been observed in such rivers (Pokrovsky & Schott, 2002). Cerium is another metal that appears to be associated with Al and Fe concentrations in freshwater, having been observed to be controlled by pH and DOC concentrations and to exhibit strong correlations with Fe and Al (along with the other rare earth metals; Gaillardet et al., 2003; Neal, 2005).

The concentrations of these metals (Al, Fe, Ti, and Ce) in stream water appear to be controlled by similar processes, and thus they are highly inter‐related. The dissolved fraction of each of these metals is correlated specifically with the cationic fraction of Al (Al_i_; Table 5), meaning we can use the more commonly measured dissolved metal concentrations to predict the rarely measured speciated forms of Al.

**Table 5:** Posterior medians of the standardized linear regression coefficients and their 95% credible intervals (2.5th–97.5th percentiles; Q)

Predictor	Median (Q_50_)	Q_2.5_	Q_97.5_
Al_d_ (μg/L)	0.79	0.65	0.93
DOC (mg/L)	−0.33	−0.49	−0.18
Ti_d_ (μg/L)	0.20	0.07	0.28
Fe_d_ (μg/L)	0.16	0.03	0.27
F (μg/L)	−0.11	−0.23	−0.02
*T*_w_ (°C)	0.10	0.05	0.16
pH	0.08	0.02	0.14
Ce_d_ (μg/L)	−0.07	−0.17	0.01
Alkalinity (mg CaCO_3_/L)	−0.06	−0.12	−0.02
Li_d_ (μg/L)	0.05	−0.02	0.13
SO_4_ (mg/L)	0.04	−0.08	0.15
Ca_d_ (mg/L)	0.03	−0.09	0.19
Color	−0.01	−0.11	0.10

DOC = dissolved organic carbon; *T*_w_ = water temperature.

Previous modeling research conducted with a smaller subset of the data set we used also found that *T*_w_ was one of the most important predictors of Al_i_ concentrations. Previous studies (Hendershot et al., 1986; Sterling et al., 2020) hypothesized that this positive relationship was caused by the role that increased temperature plays in activating biological drivers that mobilize Al (Gensemer & Playle, 1999; Santore et al., 2018). In the present study, as in other studies, we observed a statistically significant negative correlation between pH and Al_i_ concentrations, which agrees with our knowledge of how Al speciation varies with pH, although Sterling et al. (2020) did not find a significant relationship between pH and Al_i_ concentrations (Campbell et al., 1992; DeForest et al., 2018; Gensemer et al., 2018; Helliweli et al., 1983; Kopáček et al., 2006; Kroglund et al., 2001; Lydersen, 1990; Seip et al., 1989; Teien et al., 2006).

The observed negative relationship between SO_4_ and Al_i_ concentrations was unexpected, because SO_4_ is a product of acid deposition and can be considered an indicator of acidification status (see Driscoll & Wang, 2019; Strock et al., 2014). There is also a significant positive relationship between SO_4_ and pH across all sites in our data set. Most of the SO_4_ concentrations at our study sites are very low (mean concentration of 2.3 mg/L across the entire data set), and the observed relationships indicate that SO_4_ is not a major contributor to the acidity at these sites.

Our results are also consistent with previous research on Al dynamics showing a phenomenon of “decoupling” between base cation and Al concentrations when base cations were extremely low (Ca concentrations below 1.4 mg/L; Rotteveel & Sterling, 2020). We hypothesize that the strong positive relationship between Al_i_ and Ca_d_ concentrations observed at the large‐sample study sites is due to their extremely low Ca_d_ concentrations (mean = 1.1 mg/L, 75% of observations <1.3 mg/L) leading to this phenomenon of decoupling. Within the larger data set, in which the Ca_d_ concentrations were slightly higher (mean = 1.8 mg/L), the relationship between Al_i_ and Ca_*d*_ was negative.

### Using drivers to create a predictive model for Al_i_ concentrations

**Figure 5:**
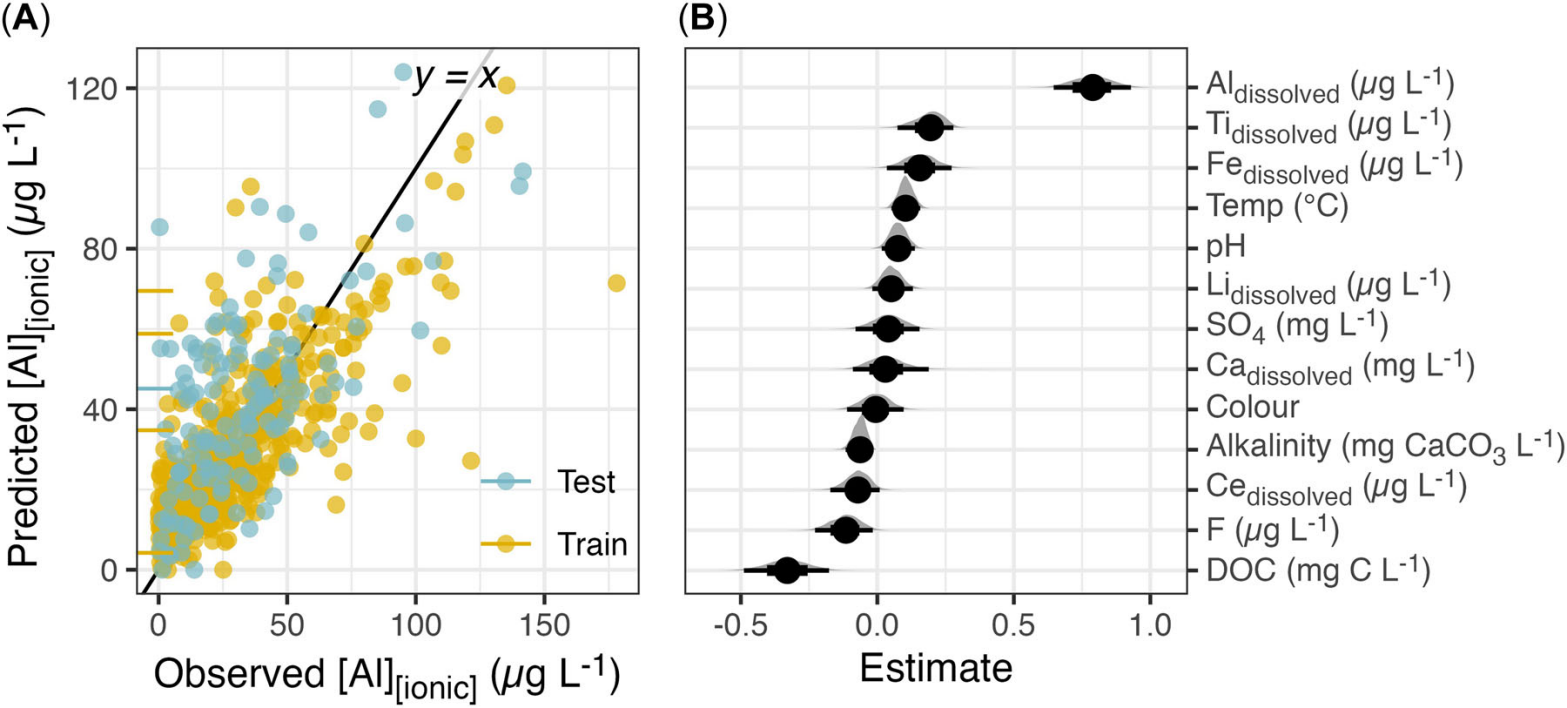
(**A**) Observed Al_i_ concentrations and corresponding model predictions in the training and test sets. Horizontal line segments represent left‐censored values; they extend from the left edge of the plot to the censoring limit. (**B**) Posterior probabilities of the regression coefficients; points represent posterior medians, and heavy and light horizontal lines span the middle 66% and 95% of the posterior distributions, respectively.

The strongest BLMM predictors of Al_i_ concentrations were Al_d_, DOC, Ti_d_, Fe_d_, and F concentrations (Figure 5 and Table 5). Dissolved Al has the largest standardized coefficient, but other predictors—particularly DOC and dissolved Ti—are influential. These results are consistent with previous modeling studies using WHAM, which found the most important variables to be Al, pH, DOC, F, Fe, Ca, and Mg; Cory et al., 2007).

**Figure 6:**
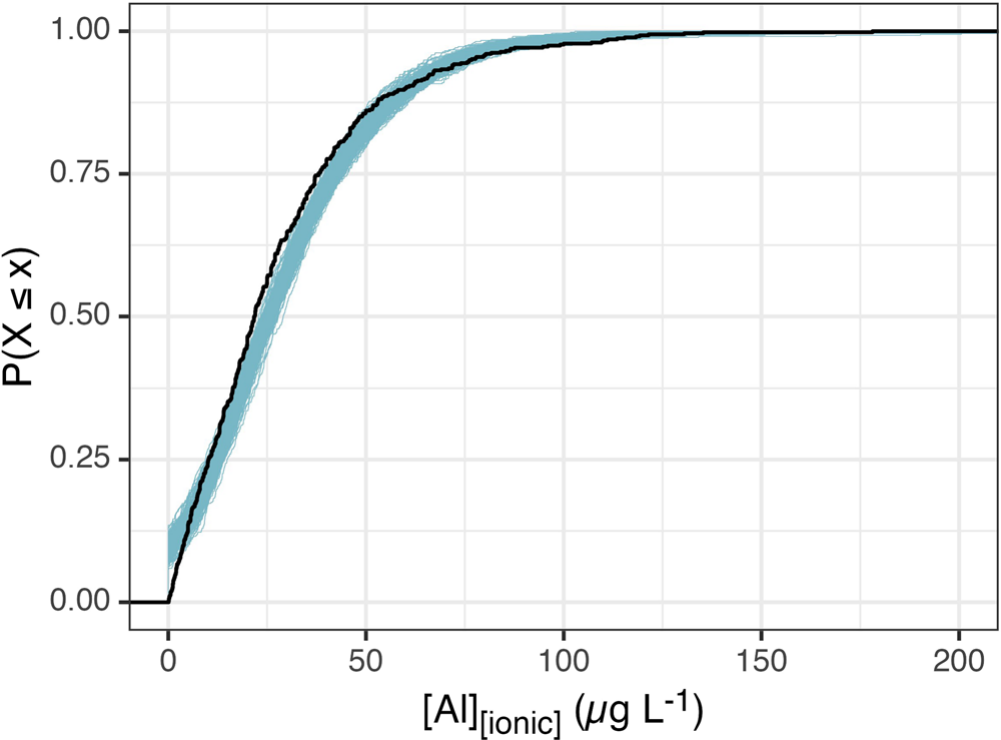
Empirical cumulative distribution functions of the observed Al_i_ concentrations and of a set of 100 posterior predictions. The Al_i_ concentrations are overpredicted at low concentrations and underpredicted at high concentrations.

The median absolute error of training set predictions was 8 μg/L, with a 95% credible interval of 6.9 to 8.2 μg/L. The median absolute error of test set predictions, used to simulate prediction of future observations, was 13 μg/L, with a 95% credible interval of 11.9 to 15 μg/L. Cumulative distribution functions show that the model slightly overpredicts Al_i_ at low concentrations and slightly underpredicts Al_i_ at high concentrations (Figure 6). Pearson correlations among posterior draws representing the Al_i_ model coefficients are visualized in the Supporting Information, Figure S1, in Appendix B.

Of note is that within the BLMM, once all other variables were accounted for, the relationship between DOC and Al_i_ switched to a negative correlation, as opposed to the positive correlation observed in the independent linear correlations. Dissolved organic carbon can form nonbioavailable complexes with Al, which may be a possible explanation for the negative relationship (Driscoll & Schecher, 1990). The appearance of F as a strong predictor of Al_i_ concentrations in the model is also of interest, given that there was no significant relationship between F and Al_i_ concentrations in the independent linear correlations. Previous studies have observed F concentrations to have a significant impact on Al abundance and speciation; however, the negative relationship we observed was unexpected because F typically forms inorganic complexes with Al and has been observed to increase the proportion of Al_i_ in freshwater (Berger et al., 2015). This positive relationship was observed to occur at high concentrations of F (median ~ 1 mg/L), while the average concentration of F in our data set was only 0.03 mg/L.

The BLMM can be used to predict Al_i_ concentrations when it is not directly monitored using more commonly measured parameters to within 15 μg/L. As when using any modeled values, caution must be taken when applying predictions from this model, which, furthermore, is not intended for causal inference. Due to the highly variable nature of Al chemistry and its many interactions with other water chemistry parameters, the BLMM will likely perform most effectively when used to make predictions in rivers with similar chemistry to those in our data set, that is, acidic (mean pH of 4.9 across the data set), dilute (mean SPC of 38 μS/cm), and with relatively high levels of organic acidity (mean DOC concentration of 11 mg/L). These conditions are common and widespread in Nova Scotia due to the region's unique combination of historic heavy acid deposition, low‐buffering bedrock that is slow‐weathering and nutrient‐poor, and high organic acidity from wetlands. The model we present is curated specially to handle these conditions, while previous more comprehensive models may lose accuracy in extreme conditions such as low pH or high Al_i_ concentrations (see Cory et al., 2007; Sjöstedt et al., 2010). To further test the model, we recommend future additional sampling of rivers that do not meet these specific conditions, to test how robust this model to a wider variation in key explanatory variables.

The BLMM may be limited by the distribution of samples collected throughout the year. Sample collection dates are heavily skewed toward the spring months of April and May, with a smaller but even number of samples collected during the summer and fall, and very few samples collected during the winter months (Supporting Information, Figure S2). Previous study of Al chemistry in Nova Scotia has shown that Al dynamics vary seasonally (Rotteveel & Sterling, 2020), so the data set we used may be missing a portion of the Al picture and should likely not be used for making winter predictions.

Discharge (i.e., base flow vs. high flow) plays an important role in water chemistry, but we unfortunately did not have access to consistent discharge measurements for our sites during the study period. Because we did not have discharge data, the concentration data are raw (not discharge corrected), meaning we are not able to detect biases related to hydrograph position. We strongly recommend that any future modeling study include the collection of discharge data and the stratification of water chemistry data by flow conditions to improve results.

An additional limitation of our study is the length of the data record. Although we did observe significant trends, they are limited due to the brevity of the data record (6 years). This may also affect the certainty of the correlations that we found. We recommend extended monitoring periods for future research.

Although complex chemical equilibrium models are important tools, the empirical model we present is relatively simple to understand and use with previously collected water chemistry data and could make modeling of Al_i_ concentrations more widely accessible to groups such as community watershed organizations. It is unrealistic for these groups, as well as government branches and academic researchers, to include speciated Al analysis in their regular sampling programs, but this model could be used to prioritize sites and regions where this type of sampling should be undertaken. This will be especially important in chronically acidified regions such as Nova Scotia, where toxic concentrations of Al_i_ may be widespread.

## Supporting Information

The Supporting Information is available on the Wiley Online Library at https://doi.org/10.1002/etc.5997.

## Conflict of Interest

The authors declare no conflict of interest.

## Author Contributions Statement

**Kristin A. Hart**: Data curation; Formal analysis; Methodology; Project administration; Software; Supervision; Visualization; Writing—original draft; Writing—review & editing. **Benjamin Trueman**: Formal analysis; Methodology; Software; Validation; Visualization, Writing—original draft; Writing—review & editing. **Edmund A. Halfyard**: Conceptualization; Funding acquisition; Methodology. **Shannon M. Sterling**: Conceptualization; Funding acquisition; Methodology; Project administration; Supervision; Writing—review & editing.

## Supporting information

This article includes online‐only Supporting Information.

Supplementary information.

Supplementary information.

## Data Availability

The code and data necessary to reproduce the model and its predictions are available at github.com/bentrueman/al-i-prediction.
