# Supplementary material for: Detection and Prediction of Toxic Aluminum Concentrations in High‐Priority Salmon Rivers in Nova Scotia
Source: Environ Toxicol Chem. 2024 Oct 1;43(12):2545–56. doi: 10.1002/etc.5997 (PMC11619745; doi:10.1002/etc.5997)
Supplement: Supplementary file 1 — Supplementary information. [file ETC-43-2545-s002.docx]

Appendix A – Laboratory Analysis Methods

Metals concentrations were analyzed using the Thermo Scientific iCAP-RQ ICPMS, following the USEPA method 200.8 for the determination of trace elements in waters and wastes by inductively coupled plasma-mass spectrometry (US EPA. 1994).

Dissolved organic carbon (DOC) concentration was analyzed using the Shimadzu TOC-V TOC Analyzer, following standard method 5310 for total organic carbon (Standard Methods Committee of the American Public Health Association, American Water Works Association, and Water Environment Federation). Samples were passed through a 0.45-µm-pore-diameter filter to obtain the DOC fraction.

Concentrations of anions such as sulfate and fluoride were analyzed using the Thermo Scientific Dionex Aquion IC, following the USEPA method 300.0 for the determination of inorganic anions by ion chromatography (Pfaff 1996).

Colour was analyzed using the Hach DR6000 Spectrophotometer, following standard method 2120 for colour (Standard Methods Committee of the American Public Health Association, American Water Works Association, and Water Environment Federation).

Alkalinity was analyzed using the Mantech Autotitrator, following standard method 2320 for alkalinity (Standard Methods Committee of the American Public Health Association, American Water Works Association, and Water Environment Federation). The sub-procedure for potentiometric titration of low alkalinity was used.

# References

Pfaff JD. 1996. DETERMINATION OF INORGANIC ANIONS BY ION CHROMATOGRAPHY. In: Methods for the Determination of Metals in Environmental Samples. Elsevier. p. 388–417. [accessed 2024 Jun 27]. https://linkinghub.elsevier.com/retrieve/pii/B9780815513988500227.

Standard Methods Committee of the American Public Health Association, American Water Works Association, and Water Environment Federation. 5310 total organic carbon. In: Lipps W, Baxter T, Braun-Howland E, editors. Standard Methods For the Examination of Water and Wastewater. Washington, D.C.: APHA Press.

Standard Methods Committee of the American Public Health Association, American Water Works Association, and Water Environment Federation. 2120 color. In: Lipps W, Baxter T, Braun-Howland E, editors. Standard Methods For the Examination of Water and Wastewater. Washington, D.C.: APHA Press.

Standard Methods Committee of the American Public Health Association, American Water Works Association, and Water Environment Federation. 2320 alkalinity. In: Lipps W, Baxter T, Braun-Howland E, editors. Standard Methods For the Examination of Water and Wastewater. Washington, D.C.

US EPA. 1994. “Method 200.8: Determination of Trace Elements in Waters and Wastes by Inductively Coupled Plasma-Mass Spectrometry,” Revision 5.4. (Journal Article).
