# Supplementary material for: Detection and Prediction of Toxic Aluminum Concentrations in High‐Priority Salmon Rivers in Nova Scotia
Source: Environ Toxicol Chem. 2024 Oct 1;43(12):2545–56. doi: 10.1002/etc.5997 (PMC11619745; doi:10.1002/etc.5997)
Supplement: Supplementary file 2 — Supplementary information. [file ETC-43-2545-s001.docx]

Appendix B – Supplementary Figures

Figure S1. Pearson correlations among posterior draws representing the Al_i_ model coefficients.


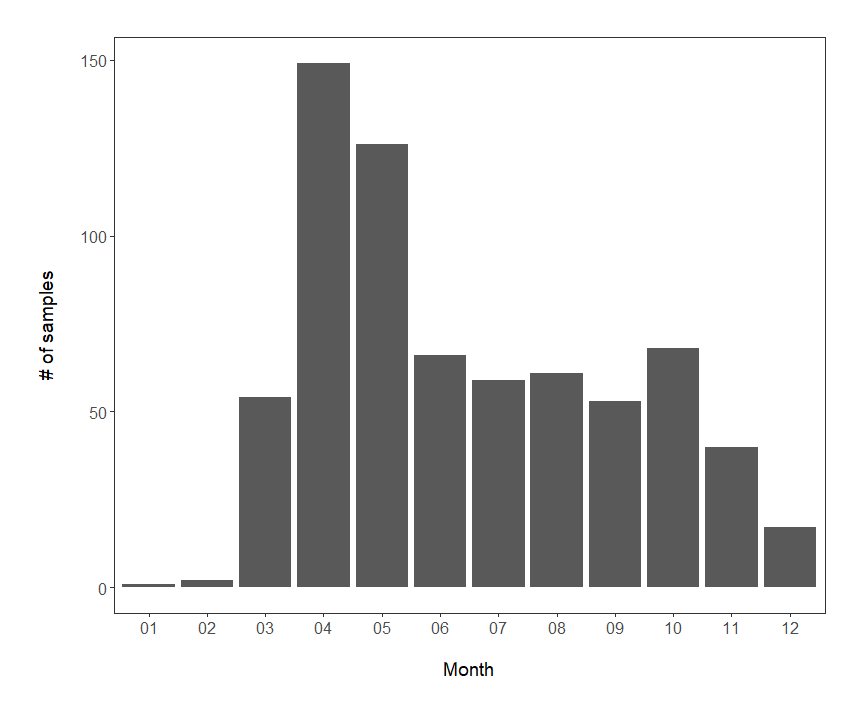


Figure S2. Distribution of samples per month over the sampling period for all sites (2015-2022).


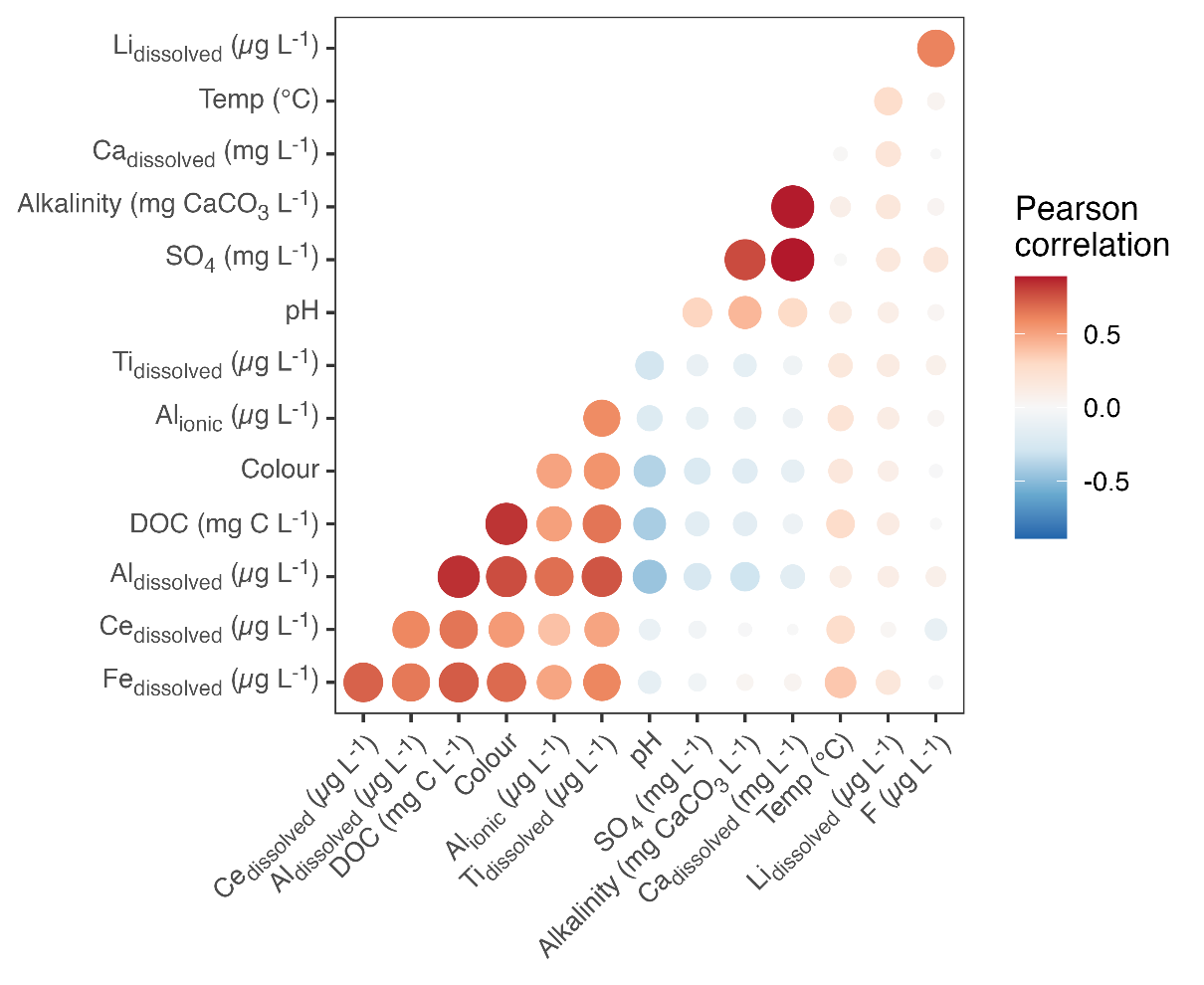


Figure S3. Pearson correlations among the variables included in the Al_i_ model.
